# Optimal strategies for COVID-19 prevention from global evidence achieved through social distancing, stay at home, travel restriction and lockdown: a systematic review

**DOI:** 10.1186/s13690-021-00663-8

**Published:** 2021-08-21

**Authors:** Tadele Girum, Kifle Lentiro, Mulugeta Geremew, Biru Migora, Sisay Shewamare, Mulugeta Shegaze Shimbre

**Affiliations:** 1 Department of Public Health, College of Medicine and Health Sciences, Wolkite University, Wolkite City, Ethiopia; 2 Department of Statistics, College of Natural and Computational Sciences, Wolkite University, Wolkite City, Ethiopia; 3 Department of Physics, College of Natural and Computational Sciences, Wolkite University, Wolkite City, Ethiopia; 4 School of Public Health, College of Medicine and Health Sciences, Arba Minch University, Arba Minch, City, Ethiopia

**Keywords:** COVID-19, Social distancing, Stay at home, Travel restriction and lockdown

## Abstract

**Background:**

Coronavirus disease (COVID-19) is a global public health agenda with high level of pandemicity. There is no effective treatment, but prevention strategies can alter the pandemic. However, the effectiveness of existing preventive measures and strategies is inconclusive. Therefore, this study aimed to review evidence related to COVID-19 prevention achieved through social distancing, stay at home, travel ban and lockdown in order to determine best practices.

**Methods/design:**

This review has been conducted in accordance with the PRISMA and Cochrane guideline. A systematic literature search of articles archived from major medical databases (MEDLINE, SCOPUS, CINAHL, PsycINFO, and Web of Science) and Google scholar was done. Observational and modeling researches published to date with information on COVID-19 prevention like social distancing, stay at home, travel ban and lockdown were included. The articles were screened by two experts. Risk of bias of included studies was assessed through ROBINS-I tool and the certainty of evidence was graded using the GRADE approach for the main outcomes. The findings were presented by narration and in tabular form.

**Results:**

A total of 25 studies was included in the review. The studies consistently reported the benefit of social distancing, stay at home, travel restriction and lockdown measures. Mandatory social distancing reduced the daily growth rate by 9.1%, contacts by 7–9 folds, median number of infections by 92% and epidemic resolved in day 90. Travel restriction and lockdown averted 70.5% of exported cases in china and doubling time was increased from 2 to 4 days. It reduced contacts by 80% and decreased the initial R_0_, and the number of infected individuals decreased by 91.14%. Stay at home was associated with a 48.6 and 59.8% reduction in weekly morbidity and fatality. Obligatory, long term and early initiated programs were more effective.

**Conclusion:**

Social distancing, stay at home, travel restriction and lockdown are effective to COVID-19 prevention. The strategies need to be obligatory, initiated early, implemented in large scale, and for a longer period of time. Combinations of the programs are more effective. However, the income of individuals should be guaranteed and supported.

## Background

The emerging Coronavirus disease 2019 (COVID-19) is becoming a global public health agenda with high level of infectiousness and mortality [1–3]. It is caused by severe acute respiratory syndrome coronavirus 2 (SARS-CoV-2) [2]. The disease novel coronavirus (formerly) was first identified in December 2019 in Wuhan, China, and has since spread globally and become a global pandemic [3–6]. The disease spread rapidly around the world, nearing 6 million confirmed cases and hundreds of thousands of deaths reported within a few months [5, 6]. Thirteen percent of the closed cohort and 2–5% of the total cohort were reportedly dead [5–9].

The full spectrum of COVID-19 ranges from subclinical infection to severe illnesses. More than 80% of cases remain asymptomatic and 15% of cases present with mild, self-limiting respiratory tract illness. While, the remaining 5% of individuals present with severe and complicated conditions such as: pneumonia, multi-organ failure, and death [1–3, 6–8]. Both asymptomatic and symptomatic cases can easily transmit the disease through direct and indirect contacts. Person-to-person transmissions primarily occur during close contact and with contaminated objects. It is most contagious during the first 3 days after the onset of symptoms [2, 7–10].

Different countries around the world have taken different preventive measures to try and keep the pandemic under public health control. Most countries implemented either of the following general strategies: complete or partial lockdown, travel ban, maintaining social distancing, frequent hand washing, maintaining physical distance, quarantine, covering coughs, and avoiding contamination of face with unwashed hands [1, 2, 7]. While, others were implemented none of these interventions or implemented in different ways [2–6]. Yet, the most efficient method is unclear [3–7].

Most of the recommended measures designed to prevent the infection were based on recommendations from researches conducted for SARS and MERS. Also the implementation strategies were based on the economic capacity of the specific country and the extent of the epidemic. This means, different countries implemented different preventive strategies differently. There is limited evidence on the effectiveness of these interventions implemented in different settings, in which the effect is not researched well [6–9].

There are a limited number of studies and to the extent of our search there is no a conclusive systematic review on the preventive aspects and effectiveness of COVID-19 infection through social distancing, stay at home, travel ban and lockdown strategies. The findings were inconclusive, in some studies certain prevention mechanisms shown to have minimal effects, while in other studies different preventive mechanisms have better effect than expected particularly for social distancing. On the other hand, some studies have reported that, integration of interventions is more effective than specific prevention strategies [1, 2, 4, 9].

Therefore, we aimed to conduct a comprehensive systematic review to determine the optimal preventive strategies achieved through social distancing, stay at home, travel ban and lockdown strategies. Hence, the synthesized analysis will be important to bring conclusive evidence. Hence, policy makers and other stakeholder will have clear evidence to make decisions in the preventive strategies of COVID-19 at the local and national context.

### Objectives

To bring optimal evidence that can support the local and national COVID-19 prevention program, through a systematic review of researches conducted on evaluation of global strategies for COVID-19 prevention through social distancing, stay at home, travel ban and lockdown measures. We aimed to answer issues related to strategic implementation and effectiveness in the prevention of the disease or death. The following key questions were considered:

- What is the optimal strategy in the implementation of social distancing, stay at home, travel ban and lockdown measures in different settings?
- Are social distancing, stay at home, travel ban and lockdown measures effective to control the COVID-19 outbreak?
- How and when these strategies should be applied to control the COVID-19 outbreak?

## Methods and materials

The reviews were conducted in accordance with the PRISMA (Preferred Reporting Items for Systematic Reviews and Meta-Analyses: guidance for reporting of systematic reviews and Meta analyses) [11] and Cochrane hand book of systematic review [12] through a systematic literature search of articles published to date. We used researches conducted throughout the glob containing information on COVID-19 prevention through social distancing, stay at home, travel ban and lockdown. The review was conducted in accordance with the protocol developed prior to the actual research. However, the protocol was not published. We gave more emphasis on the publication of the research as it is important for designing interventions.

### Eligibility criteria for the review

Researches conducted to assess the effectiveness of social distancing, stay at home, travel ban and lockdown measures for the prevention of COVID-19 were selected based on their evidence of reported outcomes relevance for decision making at local, national and international level.

#### Types of studies

In these reviews we included non-randomized observational studies conducted on COVID-19 prevention. In addition, we also included modelling (mathematical and/or epidemiological) studies, to supplement the existing evidence, as researches conducted on COVID-19 prevention are very limited.

We included Cohort studies, Case-control studies, Time series, Case series and Mathematical modelling studies conducted anywhere, any area and any setting reported in English language. Whereas; commentaries, letter to editor, case reports and governmental reports were excluded.

#### Types of participants

The participants were loosely selected. For each prevention method different participants were included. These include: individuals who have contact a confirmed or suspected case of COVID-19, or individuals who live in areas with COVID-19 outbreak; or individuals considered to be high risk for COVID-19/suspected cases, or confirmed/probable cases of COVID-19 infection. The number of participants varies according to the individual researches. We excluded individuals who have other symptomatic respiratory disease confirmed by tests.

#### Types of interventions

We included different types of interventions including: social distancing, stay at home, travel ban and lockdown measures for COVID-19 prevention applied specifically or in combination, either voluntary or mandatory and in different settings, either at a facility or in the community. In comparative studies the intervention were compared with the non-applied groups or other comparison groups. We excluded preventive interventions other than social distancing, stay at home, travel ban and lockdown measures.

#### Types of outcome measures

to decide whether a certain measure is optimal or effective for COVID-19 prevention, we used effectiveness measurements applied at different settings including: effect on incidence, disease burden, mortality reduction and epidemic control. We did not address secondary outcomes such as psychological impact, economic impact and social impact.

### Literature search strategy

We searched the MEDLINE, SCOPUS, CINAHL, PsycINFO, and Web of Science databases for studies published to date. Articles containing information on different prevention strategies (social distancing, stay at home, travel ban and lockdown) and studies assessing their effectiveness were retained for the review. A combination of free-text search terms, Medical Subject Headings, and database-specific subject headings search strategy was used for multiple electronic databases. In addition, we searched gray literatures, pre-prints and coronavirus resource centers and reference lists of systematic reviews were screened for additional relevant citations. The combination of search terms was used with (AND, OR, NOT) Boolean (Search) Operators.

1. Coronavirus Infections
2. SARS COv2
3. COVID-19
4. Novel corona
5. Prevention/ control
6. Social distancing
7. Stay home/stay at home
8. Travel bans/restriction
9. Lockdown Boundary control
10. 1 or 2 or 3 or 4 or 5 and 6 and 7 or 8 or 9 or 10

The search operation used in the Medline.

#1 exp. coronavirus/

#2 ((corona* or CORONA* or SARS*) adj1 (virus* or viral* or virinae*)).ti,ab,kw.

#3 (coronavirus* or beta-coronavirus* or coronovir* or coronavirinae* or Coronavirus* or Coronovirus* or Wuhan* or Hubei* or Huanan or “2019-nCoV” or 2019nCoV or nCoV2019 or “nCoV-2019” or “COVID-19” or COVID19 or Ncov or “n-cov” or “SARS-CoV-2” or “SARSCoV-2” or “SARSCoV2” or “SARS-CoV2” or SARSCov19 or “SARS-Cov19” or “SARSCov-19” or “SARS-Cov-19” or “middle east respiratory syndrome” or “middle-east respiratory syndrome” or Ncovor or Ncorona* or Ncorono* or NcovWuhan* or NcovHubei* or NcovChina* or NcovChinese*).ti,ab,kw.

#4 (((coronavirus* adj2 (prevention* or control*)) or “socialdistancing*” or “lockdown*” or “travelristriction*” or stayhome*”) adj

#5 “severe acute respiratory syndrome*”.ti,ab,kw.

#6 or/1–5

#7 limit 5 to yr = “2019 -Current”

### Data collection and analysis

#### Study selection process

Team of researchers (TG, MG, KL, BM, SS and MS) screened all titles and abstracts based on predefined eligibility criteria set at the protocol. Two authors (TG and MG) among the team independently screened the titles and abstracts of records retrieved during the initial search, and decided by consensus or by involving third author (MS) whenever agreements were not reached. After that, the review team (TG, MG, KL, BM, SS and MS) retrieved the full texts of all included abstracts. Two review authors (TG and MG) screened all full-text publications independently decisions were reached by consensus or by involving a third review author (MS).

#### Data extraction and management

Titles and abstracts retained from the primary electronic search were thoroughly assessed for possibility of reporting the intended outcome and for eligibility. Two authors (TG and MG) have extracted data from the included studies into standardized tables and a third author (KL) has checked the data for completeness and correctness based on the pre-sated eligibility criteria. Finally, from the retained researches the necessary information was extracted based on the structured format which includes: author, title, study participants, study design, sample size, study setting, type of intervention, length of intervention, year of publication, effect of intervention measures, type of model (for modeling studies) and results or main outcomes.

#### Quality assessment (risk of bias) in included studies

The quality and risk of bias in included studies was assessed through the Risk Of Bias In Non-randomized Studies - of Interventions (ROBINS-I) tool [13]. The first author (TG) rated the risk of bias for each study; the second author (MG) checked the ratings and third author (KL) was consulted to solve disagreements. For each study; the study design, study participants, the outcome, the presence of bias was assessed based on the eligibility criteria and quality assessment checklist. On the other hand, modelling studies were assessed by the best practice recommendations of the International Society for Pharmaco-economics and Outcomes (ISPOR) and the Society for Medical Decision making (SMDM) for dynamic mathematical transmission models tools [14].

#### Data synthesis and analysis

The qualitative part was systematically reviewed and presented in accordance with the Cochrane guideline. We synthesized results of quantitative measures narratively and in tabular form. Because of the heterogeneity of available primary studies, we did not consider quantitative analyses (meta-analysis).

#### Certainty of the evidence evaluation

The certainty of evidence was assessed using the GRADE approach [15] for the main outcomes and reported in standard terms using tables. One of the researchers (TG) assessed the certainty through assessments of risk of bias, indirectness, inconsistency, imprecision, and publication bias and classified in to four. A high certainty rating means the estimated effect lies close to the true effect; moderate certainty means the estimated effect is probably close to the true effect; a low certainty rating suggests that the estimated effect might substantially differ from the true effect; and very low certainty means that the estimated effect is probably markedly different from the true effect**.**

## Results

### Studies included

**Fig. 1:**
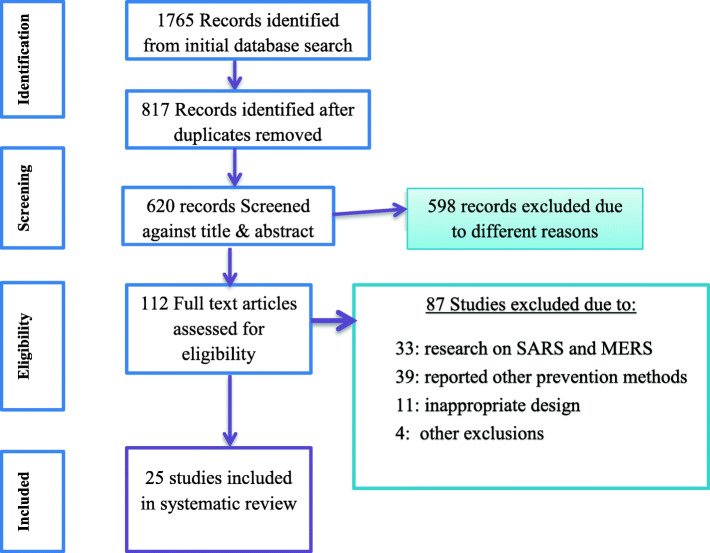
Flow chart for study search, selection and screening for the review

Figure 1 presents the PRISMA flow diagram for studies selected in the search process. Initially we identified 1765 potentially relevant citations in the form of title, abstract, bibliography and full text research from the selected databases using the electronic search system. After removal of duplicates and initial screening, 112 articles were selected for further screening and evaluation via full text. In the screening process, we found that 87 research titles were not relevant for the systematic review and removed for different reasons. 33 research was removed because the outcome was measured on SARS and MERS; 39 studies removed due to difference in intervention (prevention method other than the specified interventions), 11 studies removed due to the design difference and 4 studies removed due to other reasons. Thus, the review was conducted on 25 studies [16–40] that full filled the eligibility criteria and retained for final synthesis.

### Study characteristics

Table 1 Presents the details of **s**urvey characteristics and summary results of the included studies. The selected 25 studies [16–40] were published between January 13, 2020 and June 05, 2020. Ten of these studies [16–18, 23, 27, 28, 30, 34, 38, 39] were conducted in china, 6 in USA [20–22, 25, 31, 37], 2 in UK [32, 36], 5 globally and at least in two countries [19, 28, 29, 33, 35], and the last 3 researches conducted in Ethiopia [24], Nigeria [26] and France [40] one in each country. The included studies comprised 11 observational and event studies [16–26] and 14 modeling (SIR, SEIR and Stochastic) studies [27–40]. Twelve studies [19–23, 28–34, 36, 37] conducted on the effect of social distancing, seven studies [16–18, 26, 27, 38, 39] observed the effect of travel restriction, border control and lockdown strategies, four studies [23, 24, 35, 36] assessed non pharmacological interventions and more than one of the aforementioned prevention strategies, and the remaining one study evaluated the effect of stay at home strategy [25]. Most of these studies were community based studies and national simulation studies.

**Table 1:** Characteristics of included studies and summery of result

		Study characteristics and summery report
1	**Author/s & title[**16**]**	**Moritz U., et al. The effect of human mobility and control measures on the COVID-19 epidemic in China**
	Population size (N)	Population of China
	Country	China
	Setting	Community based
	Design	Retrospective
	Objectives	To elucidate the role of case importation in transmission in cities across China and to ascertain the impact of control measures.
	Study detail	Real-time mobility data from Wuhan and detailed case data including travel history was used to elucidate the role of case importation in transmission in cities across China and to ascertain the impact of control measures.
	Interventions	Diagnostic testing; clinical management; rapid isolation of suspected cases, confirmed cases, and contacts; and restrictions on mobility.
	Results	➢ Travel restrictions are particularly useful in the early stage of an outbreak. ➢ Travel restrictions may be less effective once the outbreak is more widespread. ➢ The combination of interventions implemented in China was clearly successful in mitigating spread and reducing local transmission of COVID-19. ➢ In this work it was not possible to definitively determine the impact of each intervention.
2	**Author/s & title[**17**]**	**Chad R. Wells, et al. Impact of international travel and border control measures on the global spread of the novel 2019 coronavirus outbreak**
	Population size (N)	Global population
	Country	China
	Setting	Community based
	Design	Retrospective
	Objectives	To estimate the impact control measures and investigate the role of the airport travel network on the global spread of the COVID-19 outbreak.
	Study detail	Daily incidence data of COVID-19 outbreak within mainland China from December 8, 2019 to February 15, 2020, as well as airline network data was used to predict the number of exported cases with and without measures of travel restriction and screening.
	Interventions	Border measures, including travel lockdown, contact tracing at the epicenter, and airport screening
	Results	➢ Travel lockdowns enforced by the Chinese government averted 70.5% of exported cases. ➢ At early stage of the epidemic, reduction in the rate of exportation could delay the importation of cases into cities unaffected by the COVID-19 outbreak, buying time to coordinate an appropriate public health response.
3	**Author/s & title[**18**]**	**Hien Lau, et al. The positive impact of lockdown in Wuhan on containing the COVID-19 outbreak in China**
	Population size (N)	China domestic Air passengers
	Country	China
	Setting	Community based
	Design	Retrospective
	Objectives	To evaluate whether rigorous lockdown measures as implemented by China have the potential to slow down the virus’ spread.
	Study detail	China domestic air traffic and passenger data before and after the lockdown was used to evaluate whether rigorous lockdown measures as implemented by China have the potential to slow down the virus’ spread.
	Interventions	Lockdown
	Results	➢ A significant increase in doubling time from 2 days to 4 days after imposing lockdown. ➢ A further increase is detected after changing diagnostic and testing methodology to 19.3. ➢ A significantly decreased growth rate and increased doubling time of cases was observed, which is most likely due to Chinese lockdown measures.
4	**Author/s & title[**19**]**	**Steffen Juranek and Floris T. Zoutman. The effect of social distancing measures on intensive care occupancy: evidence on COVID-19 in Scandinavia**
	Population size (N)	Population of Sweden, Denmark and Norway
	Country	Sweden, Denmark and Norway
	Setting	Community based
	Design	An event study (Sweden as control group, whereas Denmark and Norway each function as a treatment group)
	Objectives	To understand the effectiveness of social distancing on the spread of COVID-19.
	Study detail	A case study was conducted to compare the effect on the health care system of the strict lockdown measures by Denmark and Norway with the more lenient approach (social distancing) of Sweden.
	Interventions	Social distancing and lockdown
	Results	➢ Without lockdown, Denmark would have seen 107% more patients in ICU at the peak, and 134% more overall hospitalizations. ➢ At the end of the sample, cumulative deaths would have been 167% higher. ➢ Following the Swedish approach would have resulted in more than twice as many hospitalizations and intensive care patients at the peak, potentially bringing Denmark and Norway close to their maximum capacity. ➢ Compared to the more lenient approach of Sweden, the lockdown measures strongly reduce the number of hospitalizations and intensive care patients per capita
5	**Author/s & title[**20**]**	**Hamada S. Badr, et al. Social distancing is effective at mitigating COVID-19 transmission in the United States**
	Population size (N)	Population of USA
	Country	USA
	Setting	Community based, health care
	Design	Retrospective
	Objectives	To represent social distancing behavior derived from mobile phone data and examine its relationship with COVID-19 case reports at the county level.
	Study detail	Epidemiological data on cases and deaths for each US state were used
	Interventions	Social distancing
	Results	➢ The effect of social distancing on decreasing transmission is not perceptible for nine to twelve days after implementation. ➢ The strong relationship between social distancing and outbreak case growth rates suggest that a return to ‘normal’ interaction patterns will result in an increase in case growth rates, which may appear 9–12 days after behavioral changes ensue. ➢ However, under these changes, additional precautions such as hand washing, wearing masks and self-isolation when sick may help to lessen the case growth rates.
6	**Author/s & title[**21**]**	**Abel Brodeur, et al. On the effects of COVID-19 safer-at-home policies on social distancing, car crashes and pollution**
	Population size (N)	USA
	Country	USA
	Setting	Community based
	Design	Retrospective
	Objectives	To understand the effects safer-at-home policies on social distancing, travel and pollution.
	Study detail	Data from reports across USA were obtained to understand the effects safer-at-home policies on social distancing, travel and pollution.
	Interventions	Safer-at-home
	Results	➢ Safer-at home policies are successful in encouraging social distance. ➢ A 50% reduction in vehicular collisions; an approximately 25% reduction in particulate matter (PM2.5) concentrations; and a reduction of the incidence of county-days with an air quality index of code yellow or above by two-thirds.
7	**Author/s & title[**22**]**	**Charles Courtemanche, et al. Strong social distancing measures in the United States reduced the COVID-19 growth rate**
	Population size (N)	Population of USA
	Country	USA
	Setting	Community based
	Design	Retrospective
	Objectives	To evaluate the impact of these measures on the growth rate of confirmed COVID-19 cases across USA.
	Study detail	An event-study regression with multiple treatments was adopted to estimate the relationship between social distancing policies and the exponential growth rate of confirmed COVID-19 cases of US obtained from March 1, 2020 to April 27, 2020, a sample size of 182,004.
	Interventions	SIPOs, public school closures, bans on large social gatherings, and closures of entertainment-related businesses.
	Results	➢ Government-imposed social distancing measures reduced the daily growth rate by 5.4 percentage points after 1–5 days, 6.8 after 6–10 days, 8.2 after 11–15 days, and 9.1 after 16–20 days. ➢ Holding the amount of voluntary social distancing constant, more than 35 times greater spread without any of the four measures (35 million)
8	Author/s & title [23]	Shanlang Lin, et al. Which measures are effective in containing COVID-19?
	Population size (N)	Population of china
	Country	China
	Setting	Community based
	Design	Retrospective
	Objectives	To theoretically explain the impact mechanism of traffic control and social distancing measures on the spread of the epidemic, and empirically tests the effect of the two measures in China at the present stage using econometric approach
	Study detail	To theoretically explain the impact mechanism of the interventions on the spread of the epidemic, and empirically tests the effect of the two measures using econometric approach based on panel data of 279 prefecture-level cities from January 1 to February 10, 2020′.
	Interventions	Traffic control and social distancing
	Results	➢ The effect of social distancing is better than traffic control. ➢ The two measures are complementary, and their combined effect will play a better role in epidemic prevention. ➢ Traffic control and social distancing do not work everywhere. ➢ Traffic control only play a role in cities with large GDP and population size, but not in cities with low GDP population size. ➢ In cities with lower population size, social distancing becomes inoperative.
9	**Author/s & title[**24**]**	**Yohannes Kebede, et al. Knowledge, perceptions and preventive practices towards COVID-19 early in the outbreak among Jimma university medical center visitors, Southwest Ethiopia**
	Population size (N)	Health center visitors
	Country	Ethiopia
	Setting	Health facility based
	Design	Cross-sectional
	Objectives	To assess knowledge, perceptions, and practices that inform communication and community engagement efforts in the fight against COVID-19 among community members who visited Jimma University Medical Center (JUMC) in Jimma town, Southwest Ethiopia.
	Study detail	A cross sectional survey was conducted in Jimma university medical center during March 22–28, 2020 based on a sample size of 247 visitors.
	Interventions	Stay home with other interventions
	Results	To protect themselves from COVID-19, 1.6% individuals started to stay home, engaged in frequent hand washing with water and soap (77.3%), stopped shaking hands while giving greeting (53.8%), avoiding physical proximity (33.6%) and avoiding going to crowed places (33.2%).
10	**Author/s & title[**25**]**	**James H. Fowler, et al. The effect of stay-at-home orders on COVID-19 cases and fatalities in the United States**
	Population size (N)	Population of USA
	Country	USA
	Setting	Community based
	Design	Retrospective
	Objectives	To estimate the effect of stay-at-home orders using a difference-in- differences design that accounts for unmeasured local variation in factors like health systems and demographics and for unmeasured temporal variation in factors like national mitigation actions and access to tests.
	Study detail	Data on stay-at-home orders, COVID-19 confirmed cases, rates of testing, and fatalities by day and county throughout the United States were used to estimate the effect of stay-at-home orders.
	Interventions	Stay at home
	Results	➢ Stay-at-home associated with a 30.2% (11.0 to 45.2) reduction in weekly cases after one week, a 40.0% (23.4 to 53.0) reduction after two weeks, and a 48.6% (31.1 to 61.7) reduction after three weeks. ➢ Stay-at-home orders are also associated with a 59.8% (18.3 to 80.2) reduction in weekly fatalities after three weeks. ➢ Stay-at-home orders reduced confirmed cases by 390,000 (170,000 to 680,000) and fatalities by 41,000 (27,000 to 59,000) within the first three weeks in localities where they were implemented.
11	**Author/s & title[**26**]**	**Ekienabor Ehijiele. Coronavirus (Covid-19): the lockdown strategy in Nigeria**
	Population size (N)	Population of Nigeria
	Country	Nigeria
	Setting	Community based
	Design	Retrospective
	Objectives	To investigate the effect of the lockdown strategy in curbing the spread of the COVID-19 virus in Nigeria.
	Study detail	Data obtained from secondary sources were used to investigate the effect of the lockdown strategy in curbing the spread of the COVID-19 virus in Nigeria.
	Interventions	Lockdown
	Results	➢ Daily relative risk increases in cases, and daily relative risk increase in mortality. ➢ The observed growth in cases where in areas where active measures were not taken. ➢ Halt in business activities has rendered many penniless and unable to provide for themselves basic amenities. ➢ There is need to implement community-level measures of social distancing which may include closing schools, need for individuals with COVID-19 case or respiratory symptoms be properly taken care of, trace and quarantine those who must have come in contact with affected persons and introducing stay at home palliatives for the general public.
12	**Author/s & title[**27**]**	**Matteo Chinazzi, et al. The effect of travel restrictions on the spread of the 2019 novel coronavirus (COVID-19) outbreak**
	Population size (N)	Global Population
	Country	China
	Setting	Community based
	Design	Global metapopulation disease transmission modeling
	Objectives	To project the impact of travel limitations on the national and international spread of the epidemic.
	Study detail	Global metapopulation disease transmission model was used to project the impact of travel ban on the basis of internationally reported cases.
	Interventions	Travel restrictions/ban
	Results	➢ The travel quarantine of Wuhan delayed the overall epidemic progression by only 3 to 5 days in mainland China but had a more marked effect on the international scale, where case importations were reduced by nearly 80% until mid-February. ➢ Sustained 90% travel restrictions to and from mainland China only modestly affect the epidemic trajectory unless combined with a 50% or higher reduction of transmission in the community.
13	**Author/s & title[**28**]**	**Alexander Chudik, et al. Voluntary and mandatory social distancing: evidence on COVID-19 exposure rates from Chinese provinces and selected countries**
	Population size (N)	China and some European countries
	Country	China and some European countries
	Setting	Community based
	Design	Mathematical Modeling, SIR
	Objectives	To evaluate social distancing polices impact on both the COVID-19 epidemic and the associated employment costs.
	Study detail	A standard SIR model was fitted in order to evaluate the impact of alternative mitigation or containment policies on both the epidemic and the so-called recession curves, and to empirically compare their implementation across countries.
	Interventions	Voluntary and mandatory social distancing
	Results	➢ Mandated policies can be very useful in fattening the epidemic curve, but is costly in terms of employment loss. ➢ Voluntary policies are relatively ineffective. ➢ Self-isolation can affect the epidemic curve, but only when it is close to its peak.
14	**Author/s & title[**29**]**	**William Broniec, et al. Using VERA to explain the impact of social distancing on the spread of COVID-19**
	Population size (N)	General population
	Country	Global
	Setting	Community based/Simulation
	Design	VERA_Epidemiology, SIR Modeling
	Objectives	To describe the use of VERA to develop SIR model for the spread of COVID-19 and its relationship with healthcare capacity.
	Study detail	SIR model was developed based on VERA to express the impact of social distancing on the spread of the disease but also the management of the impact of the disease on the healthcare capacity based on sample population of 10,000 people
	Interventions	Social distancing
	Results	➢ Simulation results with 16 average contacts per day per person, the number of infected individuals exceeds the healthcare capacity of the system very early under these conditions. ➢ Simulation results with 12 average contacts per day per person, the number of infected individuals exceeds the healthcare capacity of the system after 20 days under these conditions. ➢ Reducing the Average contacts per day per person hypothetically suggest that people are reducing social contact somewhat, but not substantially.
15	**Author/s & title[**30**]**	**Juanjuan Zhang, et al. Age profile of susceptibility, mixing, and social distancing shape the dynamics of the novel coronavirus disease 2019 outbreak in China**
	Population size (N)	Wuhan and Shanghai
	Country	China
	Setting	Community based
	Design	SIR modeling
	Objectives	To disentangle how transmission is affected by age differences in the biology of COVID-19 infection and disease, and altered mixing patterns due to social distancing.
	Study detail	Contact surveys data for Wuhan and Shanghai before and during the outbreak were analyzed using SIR model.
	Interventions	Social distancing
	Results	➢ Daily contacts were reduced 7–9 fold during the COVID-19 social distancing period, with most interactions restricted to the household. ➢ Social distancing alone, as implemented in China during the outbreak, is sufficient to control COVID-19. ➢ While proactive school closures cannot interrupt transmission on their own, they reduce peak incidence by half and delay the epidemic.
16	**Author/s & title[**31**]**	**Stephen Kissler, et al. Social distancing strategies for curbing the COVID-19 epidemic**
	Population size (N)	Population of USA
	Country	USA
	Setting	Community based
	Design	Mathematical modeling, SEIR
	Objectives	To assess the amount of social distancing needed to curb the SARS-CoV-2 epidemic in the context of seasonally varying transmission.
	Study detail	SEIR model was fitted using simulated data.
	Interventions	Social distancing
	Results	➢ One-time interventions will be insufficient to maintain COVID-19 prevalence within the critical care capacity of the United States. ➢ Intermittent distancing measures can maintain control of the epidemic, but without other interventions, these measures may be necessary into 2022. ➢ Increasing critical care capacity could reduce the duration of the SARS-CoV-2 epidemic while ensuring that critically ill patients receive appropriate care.
17	**Author/s & title[**32**]**	**Bendtsen Cano, et al. COVID-19 modelling: the effects of social distancing**
	Population size (N)	60 million
	Country	UK
	Setting	Community based, simulation
	Design	Stochastic modeling
	Objectives	To represent the disease dynamics of Covi-19 and the efficacy of social distancing.
	Study detail	Markov chain model was applied to represent the disease dynamics of Covi-19 and the efficacy of social distancing based on 60 million simulation.
	Interventions	Social distancing
	Results	➢ In the case of perfect social distancing (R_0_ = 0) the mortality rate is only 0.04% (21,474 dead) and the epidemic is resolved by day 90 with the number of people sick peaking on day 70. ➢ In the case of a more relaxed social distancing with (R_0_ = 0.5) the mortality rate is 0.13% (79,781 dead) without having the epidemic resolved by day 250 and with the number of people sick peaking on day 71. ➢ A somewhat stricter social distancing with R_0_ = 0.25 would resolve the epidemic by day 189 and lead to 32,998 dead. ➢ If the social distancing is relaxed to R_0_ = 0.75 a much later peak in the number of people sick on day 112 and also a much larger mortality rate of 0.55% (330,964 dead).
18	**Author/s & title[**33**]**	**Per Block, et al. Social network-based distancing strategies to flatten the COVID-19 curve in a post-lockdown world**
	Population size (N)	General population
	Country	Global
	Setting	Community based
	Design	Stochastic simulation
	Objectives	To evaluate the effectiveness of three distancing strategies designed to keep the curve flat and aid compliance in a post-lockdown world.
	Study detail	Stochastic infection curve was simulated which incorporate core elements from infection models, ideal-type social network models and statistical relational event models.
	Interventions	Three distancing strategies (limiting interaction to a few repeated contacts akin to forming social bubbles; seeking similarity across contacts; and strengthening communities via triadic strategies)
	Results	A strategic social network-based reduction of contact strongly enhances the effectiveness of social distancing measures while keeping risks lower.
19	**Author/s & title[**34**]**	**Kiesha Prem, et al. The effect of control strategies to reduce social mixing on outcomes of the COVID-19 epidemic in Wuhan, China: a modelling study.**
	Population size (N)	Population of Wuhan
	Country	China
	Setting	Community based
	Design	SEIR modeling, case study
	Objectives	To estimate the effects of physical distancing measures on the progression of the COVID-19 epidemic.
	Study detail	A mathematical model was used to quantify the potential impacts of physical distancing policies, relying on Wuhan as a case study.
	Interventions	Physical distancing
	Results	➢ Physical distancing measures were most effective if the staggered return to work was at the beginning of April; this reduced the median number of infections by more than 92 and 24% in mid-2020 and end-2020, respectively. ➢ Effects of physical distancing measures vary by the duration of infectiousness and the role school children have in the epidemic.
20	**Author/s & title[**35**]**	**Neil M. Ferguson, et al. Impact of non-pharmaceutical interventions (NPIs) to reduce COVID-19 mortality and healthcare demand**
	Population size (N)	Population of UK and USA
	Country	UK and USA
	Setting	Community based
	Design	Mathematical modeling
	Objectives	To assess the potential role of a number of public health measures -so-called non-pharmaceutical interventions (NPIs) aimed at reducing contact rates in the population and thereby reducing transmission of the virus.
	Study detail	An individual-based simulation model which is developed to support influenza pandemic was modified to explore scenarios for COVID-19.
	Interventions	Non-pharmaceutical interventions
	Results	➢ To reduce R_0_ to close to 1 or below, a combination of case isolation, social distancing of the entire population and either household quarantine or school and university closure are required. ➢ Optimal mitigation policies (combining home isolation of suspect cases, home quarantine of those living in the same household as suspect cases, and social distancing of the elderly and others at most risk of severe disease) might reduce peak healthcare demand by 2/3 and deaths by half.
21	**Author/s & title[**36**]**	**Adam J. Kucharski, et al. Effectiveness of isolation, testing, contact tracing and physical distancing on reducing transmission of SARS-CoV-2 in different settings: a mathematical modelling study**
	Population size (N)	Population of UK
	Country	UK
	Setting	Community based
	Design	Mathematical modeling
	Objectives	To understand what combination of measures-including novel digital tracing approaches and less intensive physical distancing-may be required to reduce transmission of SARS-CoV-2.
	Study detail	Using a model of individual-level transmission stratified by setting (household, work, school, other) based on BBC pandemic data from 40,162 UK participants, the impact of a range of different testing, isolation, tracing and physical distancing scenarios were simulated.
	Interventions	Isolation, testing, contact tracing and physical distancing
	Results	➢ Combined isolation and tracing strategies would reduce transmission more than mass testing or self-isolation alone (50–60% compared to 2–30%). ➢ If limits are placed on gatherings outside of home/school/work, then manual contact tracing of acquaintances only could have a similar effect on transmission reduction as detailed contact tracing. ➢ High proportion of cases would need to self-isolate and a high proportion of their contacts to be successfully traced to ensure an effective R_0_ that is below one in the absence of other measures.
22	**Author/s & title[**37**]**	**Deanna M. Kennedy, et al. Modeling the effects of intervention strategies on COVID-19 transmission dynamics**
	Population size (N)	Population of USA
	Country	USA
	Setting	Community based
	Design	Mathematical modeling study, SUEIHCDR
	Objectives	To model the effects of continuous, intermittent, and stepping-down social distancing (SD) strategies and personal protection measures on COVID-19 transmission dynamics.
	Study detail	A SUEIHCDR model was employed to model the effects of continuous, intermittent, and stepping-down social distancing (SD) strategies and personal protection measures on COVID-19 transmission dynamics, model results were based on an average of 5000 runs/simulations.
	Interventions	Social distancing
	Results	➢ The stepping-down (SD) strategy was the best long-term SD strategy to minimize the peak number of active COVID-19 cases and associated deaths. ➢ The stepping-down strategy also resulted in a reduction in total time required to SD over a two-year period by 6.5% compared to an intermittent or constant SD strategy. ➢ An 80-day SD time-window was statistically more effective in maintaining control over the COVID-19 pandemic than a 40-day window.
23	**Author/s & title[**38**]**	**Biao Tang, et al. Estimation of the transmission risk of the 2019-nCoV and its implication for public health interventions**
	Population size (N)	Population of China
	Country	China
	Setting	Community based, health care
	Design	Mathematical Modeling, R_0_ = 6.47
	Objectives	To estimate the basic reproduction number by means of mathematical modeling for determining the potential and severity of an outbreak and providing critical information for identifying the type of disease interventions and intensity based on data obtained from laboratory confirmed Covid-19 cases in mainland china.
	Study detail	A deterministic compartmental model was devised based on the clinical progression of the disease, epidemiological status of the individuals, and intervention measures.
	Interventions	Contact tracing, quarantine and isolation
	Results	➢ Interventions, such as intensive contact tracing followed by quarantine and isolation, can effectively reduce the control reproduction number and transmission risk. ➢ With travel restriction (no imported exposed individuals to Beijing), the number of infected individuals in seven days will decrease by 91.14% in Beijing, compared with the scenario of no travel restriction.
24	**Author/s & title[**39**]**	**Zifeng Yang, et al. Modified SEIR and AI prediction of the epidemics trend of COVID-19 in China under public health interventions**
	Population size (N)	Population of china
	Country	China
	Setting	Community based
	Design	Mathematical Modeling, SEIR and an artificial intelligence (AI) approach
	Objectives	To predict the epidemic progression of COVID-19.
	Study detail	A modified susceptible-exposed-infected-removed (SEIR) epidemiological model was used that incorporates the domestic migration data before and after January 23 and the most recent COVID-19 epidemiological data to predict the epidemic progression.
	Interventions	Strict controls on travel, quarantine and extensive monitoring of suspected cases
	Results	➢ A five-day delay in implementation would have increased epidemic size in mainland China three-fold. ➢ Lifting the Hubei quarantine would lead to a second epidemic peak in Hubei province in mid- March and extend the epidemic to late April.
25	**Author/s & title[**40**]**	**Laura Di Domenico, et al. Expected impact of lockdown in Île-de-France and possible exit strategies**
	Population size (N)	Population of France
	Country	France
	Setting	Community based
	Design	A stochastic discrete age-structured epidemic modeling
	Objectives	To (i) assess the current epidemic situation, (ii) evaluate the expected impact of the lockdown implemented in France on March 17, 2020, and (iii) estimate the effectiveness of possible exit strategies.
	Study detail	A stochastic discrete age-structured epidemic model based on demographic and age profile data of France were considered.
	Interventions	Lockdown with other interventions
	Results	➢ Prior to lockdown R_0_ is estimated at 3.0 [2.8, 3.2] (95% CI) and the population infected by COVID-19 as of April 5 to be in the range 1 to 6%. ➢ The average number of contacts is predicted to be reduced by 80% during lockdown, leading to a substantial reduction of the reproductive number (R_LD_ = 0.68 [0.62–0.73]). ➢ Lifting the lockdown with no exit strategy would lead to a second wave largely overwhelming the healthcare system. ➢ Extensive case-finding, testing and isolation are required to envision social distancing strategies that gradually relax current constraints (larger fraction of individuals going back to work, progressive reopening of activities), while keeping schools closed and seniors isolated.

### Quality assessment of included studies

Presented in Tables 2 and 3 is a summary of the risk of bias assessment of included non-randomized studies and quality rating of the modelling studies respectively. All of the observational studies have moderate risk of bias in overall assessment. Whereas, twelve of the modeling studies were rated as no concerns to minor concerns and the other two studies as major concerns [29] and moderate concerns [32].

**Table 2:** Risk of bias assessment of observational studies based on ROBINS-I

Author and year	Bias due to confounding	Bias in selection of participants into the study	Bias in classification of interventions	Bias due to deviations from intended interventions	Bias due to missing data	Bias in measurement of outcomes	Bias in selection of the reported result	Overall risk of bias
Moritz 2020 [16]	Moderate	Low	Low	Low	Moderate	Moderate	Low	Moderate
Chad 2020 [17]	Moderate	Low	Low	Low	Moderate	Moderate	Low	Moderate
Hien 2020 [18]	Moderate	Low	Low	Low	Moderate	Moderate	Low	Moderate
Steffen 2020 [19]	Moderate	Low	Low	Low	Moderate	Moderate	Low	Moderate
Hamada 2020 [20]	Moderate	Moderate	Low	Low	Moderate	Moderate	Low	Moderate
Abel 2020 [21]	Moderate	Low	Low	Low	Moderate	Moderate	Low	Moderate
Charles 2020 [22]	Moderate	Low	Low	Low	Moderate	Moderate	Low	Moderate
Shanlang 2020 [23]	Moderate	Low	Low	Low	Moderate	Moderate	Low	Moderate
Yohannes 2020 [24]	Moderate	Low	Low	Low	Moderate	Moderate	Low	Moderate
James 2020 [25]	Moderate	Low	Low	Low	Moderate	Moderate	Low	Moderate
Ekienabor 2020 [26]	Moderate	Low	Low	Low	Moderate	Moderate	Moderate	Moderate

**Table 3:** Quality rating of the modeling studies based on three best practice recommendations from ISPOR

Author and year	Was the model a dynamic (transmission) model?	Did the authors conduct uncertainty analyses on key assumptions that may have had an impact of the conclusions?	Do the results provide estimates of the change in the burden of infection due to the intervention?	Quality
Matteo 2020 [27]	Yes	Yes	Yes	No concerns to minor concerns
Alexander 2020 [28]	Yes	Yes	Yes	No concerns to minor concerns
William 2020 [29]	Yes	No	No	Major concerns
Juanjuan 2020 [30]	Yes	Yes	Yes	No concerns to minor concerns
Stephen 2020 [31]	Yes	Yes	Yes	No concerns to minor concerns
Bendtsen 2020 [32]	Yes	No	Yes	Moderate concerns
Per Block 2020 [33]	Yes	Yes	Yes	No concerns to minor concerns
Kiesha 2020 [34]	Yes	Yes	Yes	No concerns to minor concerns
Neil 2020 [35]	Yes	Yes	Yes	No concerns to minor concerns
Adam 2020 [36]	Yes	Yes	Yes	No concerns to minor concerns
Deanna 2020 [37]	Yes	Yes	Yes	No concerns to minor concerns
Biao 2020 [38]	Yes	Yes	Yes	No concerns to minor concerns
Zifeng 2020 [39]	Yes	Yes	Yes	No concerns to minor concerns
Laura 2020 [40]	Yes	Yes	Yes	No concerns to minor concerns

### COVID 19 prevention strategies

#### Social distancing strategies

With duplicates we included five observational studies [19–23] and nine modeling studies [28–34, 36, 37] that assessed the effect of social distancing with or without other preventive programs. Three of the observational studies were conducted in USA [20–22], one in china [23] and the remaining one study was conducted in Scandinavia countries (Sweden, Denmark and Norway) [19]. While the modeling studies were conducted in globally, USA, China and UK.

One of the retrospective study has been conducted in USA [22] to evaluate the impact of strong social distancing measures on the growth rate of confirmed COVID-19 and found that, government-imposed social distancing measures reduced the daily growth rate by 5.4% after 1–5 days, 6.8% after 6–10 days, 8.2% after 11–15 days, and 9.1% after 16–20 days. Another study conducted in USA [20] reported that the effect of social distancing on decreasing transmission is not appreciable for nine to 12 days after implementation of social distancing; however, after 9–12 days the effect was very high.

The other three retrospective studies conducted to evaluate the effect of social distancing complimented with stay at home policy [21], traffic control [23] and lockdown policy [19] reported that, the measures enhance the effectiveness of social distancing. According to the studies, traffic control and social distancing were complementary, and their combined effect played a better role in epidemic prevention [23]. Also implementation of safer at home policies facilitate social distancing and reduce incidence of disease by two third [21]. However, these measures were not functional everywhere. Similarly, a study in Scandinavia [19] indicated that the lockdown measures strongly reduced the number of hospitalizations and intensive care patients.

The modeling studies conducted globally and in specific countries that assessed the effect of social distancing alone or integrated in reducing incidence, mortality, epidemic peak time and cost effectiveness consistently reported that social distancing is effective in all outcomes. Early initiation and large scale control measure and government initiated social controls were very effective in controlling the disease [28–34, 36, 37]. However, social distancing and travel restriction measures implemented for longer period of time may affect employment and economy of a country, hence, may not be affordable to developing countries [28].

One of the community based SIR modeling study conducted in China [30] indicated that daily contacts were reduced by 7–9 fold during the COVID-19 social distancing period. With strict policies, social distancing alone, as implemented in China during the outbreak, is sufficient to control COVID-19. Another community based stochastic modeling simulation [32] also reported that, with perfect social distancing policies the epidemic can be controlled in 90 days and mortality is reduced much more. In case, if social distancing not works and average contacts per day per person is 16, the number of infected individuals exceeds the healthcare capacity of the system very early [29].

According to the finding of Alexander Chudik et al. [28] mandated policies can be very useful in flattening the epidemic curve, but is costly and voluntary social distancing is insufficient in controlling the disease. Also one-time interventions were insufficient [31]. Hence, a strategic social network-based contact reduction is important [33]. Similarly, a community based study conducted in Wuhan, china [34] reported that physical distancing measures and work at home initiated early reduced the median number of infections by more than 92 and 24% in mid-2020 and end-2020, respectively. In addition to these finding three studies reported that implementing the program for longer period of time and integrating with other programs such as stepping down measures are very effective [34, 36, 37].

### Travel restriction and lockdown strategies

Of the nine researches [16–18, 23, 26, 27, 38–40] assessing the effect of travel ban and lockdown with or without other strategies, five are observational studies [16–18, 23, 26] conducted in China and Nigeria. Whereas, among the four modeling studies [27, 38–40] three were conducted in china and one in France. These studies consistently reported the benefit of travel restriction and lockdown strategies to control COVID 19.

One of the studies [16] assessed the effect of human mobility and control measures in china, reported that travel restrictions are particularly useful in the early stage of an outbreak before wide spread distribution of the disease. Also the combination of interventions implemented in China was successful in mitigating spread and reducing local transmission of COVID-19. Another study concluded travel lockdowns enforced by the Chinese government averted 70.5% of exported cases and it was most effective at early stage of the epidemic. From another community based retrospective study doubling time was increased from 2 days to 4 days after imposing lockdown [18]. Others also reported the positive effect of social distancing such as school and business closure and integration of all preventive measures [23, 26].

The lockdown strategy reduced contacts by 80% and decreased the initial reproductive number from 3.0 [95% CI: 2.8, 3.2] to 0.68 [95% CI: 0.62–0.73]) [40]. According to another modeling study, with travel restriction, the number of infected individuals can be decreased by 91.14% in Beijing [38]. The other two studies basically indicated the potential effect of early intervention and combination of different interventions [27, 39].

### Stay at home strategies

Three observational studies that aimed to assess the effect of stay at home measures in Ethiopia [24] and USA [21, 25] reported the benefit of stay at home measures. A study by James H [25] aimed to measure the effect of stay at home measure in USA found that, it was associated with a 30.2% reduction in weekly cases after 1 week, a 40.0% reduction after 2 weeks, and a 48.6% reduction after 3 weeks. In addition to this, stay-at-home orders were associated with a 59.8% reduction in weekly fatalities after 3 weeks, and reduced confirmed cases by 390,000 [25]. Another two studies also reported that individuals are stay at home to prevent the infection and the effect was tremendous [21, 24].

## Discussion

This study aimed to find out optimal strategies for COVID-19 prevention from global evidence achieved through social distancing, stay at home, and travel ban and lockdown measures by reviewing existing literatures. The review identified and systematically synthesized the findings of 25 studies [16–40] conducted abroad to bring the best available evidence that policy makers and implementers can use in the process of infection prevention.

The studies consistently reported the benefit of social distancing, stay at home, and travel ban and lockdown measures for the prevention of COVID-19. Social distancing measures achieved through reducing contacts can tremendously reduce the reproductive number, increases the doubling time, reduces the duration of epidemics, and decrease the incidence and associated mortality at community level and individual level. Obligatory social distancing measures that are initiated in early stages of the epidemic and implemented for longer period of time were very effective [19–23, 28–34, 36, 37]. Implementation of these strategies along with other previously identified strategies [41] can be optimally applied in different settings.

The effect of each intervention varies depending on the extent to which the intervention was applied. In intensive interventions, the effect of social distancing is evident after 12 days of implementation and the effectiveness increases by implementing for long period of time [20]. Also strengthening and mandatory implementation of social distancing measures reduced the daily growth rate by 5.4–9.1% within 20 days [22]. This evidence is in line with the finding of other reviews and modeling studies conducted to assess the effectiveness of social distancing measures in the prevention of SARS, MERS and COVID-19 [40, 42, 43]. Integration of programs such as lockdown and travel ban along with social distancing and with other interventions enhances the effectiveness of the program. Although, travel restriction and lockdown were very important measures to control the epidemic, early initiation, larger coverage and integration with other program were very important to alter the epidemic. However, intermittent social distancing measures and travel restrictions were ineffective in most of cases and in some countries they found to have a limited effect [24, 31]. In line with this review finding, a researcher [35] reported that adding social distancing of people 70 years or older for 4 months with the existing combination of case isolation and voluntary quarantine for 3 months increase the percentage of prevented death from 31 to 49%.. Thus, the combination of case isolation, household quarantine, social distancing of the entire population, and school and university closures are the most effective combinations of measures that could reduce the reproduction number close to one and effective to prevent COVID 19 [17, 35, 36].

The present systematic review indicates that social distancing, staying at home and lockdowns are very efficient to prevent COVID-19 infections. However, these strategies harm economic activities unless work is performed remotely through telecommuting and the use of robots [44, 45]. AI and robotics have been used to solve practical problems during the COVID-19 pandemic and they can also be viewed as part of the optimal strategies to prevent COVID-19 infections. Semi-autonomous robots have been utilized for the cleaning and sterilization in hospitals and to deliver food, medication and equipment. In particular, robots have been used to implement social distancing requirements in Singapore and to deliver food to residents staying at home in the UK [45]. Unfortunately, job automation and autonomous robots have a downside and can lead to mass unemployment. Therefore, Dr. Andrew Johnson and Dr. Katherine Roberto have recently suggested that an unconditional basic income (UBI) could help people financially during the pandemic, especially those who cannot work [44–47]. UBI could be an effective safety net, especially when combined with retraining programs to teach people the necessary skills to work remotely and/or from home.

On the other hand measures undertaken to close borders, restrict travels and air transportation were important to control the disease [16, 38]. Most countries throughout the world were implemented this interventions in the early phases of the epidemic and found to contributed to control the epidemic. As evidenced in china, Lockdown was averted more than two third of exported cases and the epidemic doubling time was increased [16, 17]. Also, the lockdown strategy reduced contacts by 80% and significantly decreased the initial reproductive number from 3.0 to 0.68. With all these cumulative mechanisms the numbers of infected cases were reduced by 91.14% in Beijing [38]. As evidenced from previous studies, travel restriction can reduce the number of susceptible individuals and number of newly infected cases [41].

However, this strategy can’t be implemented for longer period of time due to economic reasons. Researches indicated that implementing travel restriction and lockdown for longer period of time reduces individual’s wage, income and challenges the global economy [44, 46]. On the other hand companies that applied digital marketing were profitable [44]. Application of remote working and digital marketing assisted with artificial intelligence and robotic technologies reduced the potential economic impact of the lockdown [44, 45]. But automation of all works with these technologies reduces human retention and results in unemployment [44, 47]. Therefore the economic impact of digitalization and application of artificial intelligence and robotics for the prevention of COVID 19 remained a point of discussion and debate [44–47].

Stay at home strategy was one of the most effective and optimal strategy in the prevention of COVID 19 infection in most countries [24, 25]. It can halt the incidence and mortality by half, if implemented strictly [25]. This strategy is commonly implemented in combination with travel restriction, quarantine and isolation in order to increase the effect of prevention measures [24, 25, 36]. However, the effectiveness of stay at home strategies can be reduced by the time when it was initiated, how strict it is and how long it was in practice [24–27].

The most important challenge in implementation of stay at home strategy has been the economic burden of the program [44–47]. Unless the government is able to fulfill the basic needs, supply demands and remote working is arranged it is quite difficult to implement the program for longer period of time. In countries where digitalization is not advanced and applications of technologies were limited, the stay home program has been removed after short time [42–44]. The economic challenge ascribed to stay at home program can’t be carried by low and middle income countries. It can challenge even to the developed states [45–47].

Optimally designed strategies such as social distancing, stay at home, travel ban and lockdown measures can significantly prevent COVID-19 epidemics in different settings. Optimal implementation of these strategies includes early initiation of obligatory and large scale programs implemented for prolonged period of time. However, this optimization depends on the government’s capacity to cope the economic burden. Optimization of strategies that integrates all or some of the above strategies highly improves the effectiveness of the program. Also all the countries have been implemented different strategies, the optimization of the program and the effectiveness was different in different countries [1–3, 38–41]. Carrying the economic burden of these intervention was the main challenges and some of the challenges can be reduced by application of digital technologies, AI, robotics and by arranging remote work [44–47].

### Limitation

This review included a wide variety of study designs (observational and model studies), hence, it failed to include meta-analysis (statistical measures). Modeled studies also assume different scenarios, where it may not be true in the general cases. Also the review has included only publications reported in English language and open accesses resources. The study don’t analysed the economic burden of the selected interventions and the potential effect of implementing other strategies that optimize the prevention program such as application of AI, Robotic and digitalization.

## Conclusion and recommendation

Social distancing, stay at home, travel ban and lockdown measures are effective for COVID-19 prevention, particularly combined together. Obligatory implementation and early initiation of combination of travel restriction, lockdown, stay at home and social distancing programs, implemented for longer period of time are very effective in the prevention of COVID 19 infection. Applications of digital technologies enhance the implementation of the program. However, these strategies harm economic activities unless work is performed remotely through telecommuting and the use of robots. Unconditional basic income could be an effective safety net, especially when combined with retraining programs to teach people the necessary skills to work remotely and/or from home. Therefore, the health care system should consider high level implementation in obligatory and longtime restriction of movement in accordance with the economic stand of the specific country.

## Data Availability

Please contact author for data requests.

## Abbreviations

COVID-19: Coronavirus disease 2019
MERS: Middle East respiratory syndrome
SARS: Severe acute respiratory syndrome
PRISMA: Preferred Reporting Items for Systematic Reviews and Meta-Analyses
R0: Basic reproduction number
SEIR: Susceptible-exposed-infected-recovered
WHO: World Health Organization
